# Synthetic method of analogues for emerging infectious disease forecasting

**DOI:** 10.1371/journal.pcbi.1013203

**Published:** 2025-06-23

**Authors:** Alexander C. Murph, G. Casey Gibson, Elizabeth B. Amona, Lauren J. Beesley, Lauren A. Castro, Sara Y. Del Valle, Dave Osthus

**Affiliations:** 1 Statistical Sciences, Computer, Computational, and Statistical Sciences Division, Los Alamos National Laboratory, Los Alamos, New Mexico, United States of America; 2 Department of Statistical Sciences & Operations Research, Virginia Commonwealth University, Richmond, Virginia, United States of America; 3 Information Systems & Modeling, Analytics, Intelligence, & Technology Division, Los Alamos National Laboratory, Los Alamos, New Mexico, United States of America; Northeastern University, UNITED STATES OF AMERICA

## Abstract

The Method of Analogues (MOA) has gained popularity in the past decade for infectious disease forecasting due to its non-parametric nature. In MOA, the local behavior observed in a time series is matched to the local behaviors of several historical time series. The known values that directly follow the historical time series that best match the observed time series are used to calculate a forecast. This non-parametric approach leverages historical trends to produce forecasts without extensive parameterization, making it highly adaptable. However, MOA is limited in scenarios where historical data is sparse. This limitation was particularly evident during the early stages of the COVID-19 pandemic, where the emerging global epidemic had little-to-no historical data. In this work, we propose a new method inspired by MOA, called the *Synthetic Method of Analogues* (sMOA). sMOA replaces historical disease data with a library of *synthetic* data that describe a broad range of *possible* disease trends. This model circumvents the need to estimate explicit parameter values by instead matching segments of ongoing time series data to a comprehensive library of synthetically generated segments of time series data. We demonstrate that sMOA has competitive performance with state-of-the-art infectious disease forecasting models, out-performing 78% of models from the COVID-19 Forecasting Hub in terms of averaged Mean Absolute Error and 76% of models from the COVID-19 Forecasting Hub in terms of averaged Weighted Interval Score. Additionally, we introduce a novel uncertainty quantification methodology designed for the onset of emerging epidemics. Developing versatile approaches that do not rely on historical data and can maintain high accuracy in the face of novel pandemics is critical for enhancing public health decision-making and strengthening preparedness for future outbreaks.

## 1. Introduction

Method of Analogues (MOA) has proven to be a powerful forecasting method across several applications [1]. In [2], MOA was used to forecast influenza incidence in France from 1984 to 2002. In this application, MOA showed superior performance to linear autoregressive models, especially in cases where there was not a precise periodicity between different influenza seasons. In [3], MOA is strengthened by incorporating historic trends on climate data, since certain climate conditions are believed to affect viral survival. Outside of disease forecasting and meteorology, the principles used in MOA are seen in several other prediction tasks, such as in forecasting reservoir inflow [4] and in predicting future environmental changes using subfossil remains [5].

MOA requires a few basic ingredients: (1) an observed time series to be forecast, (2) a library of related time series assembled from historical data, and (3) a way to measure the distance between the observed time series and each time series in the library. For situations that have all three ingredients, MOA can be applied. In *emerging* disease forecasting contexts, the missing ingredient is (2): the availability of historical data specific to the disease under study. This limitation makes MOA impractical for newly emerging epidemics: such was the case in the early days of COVID-19 forecasting. However, as historical data eventually accumulated, MOA approaches were developed and applied for COVID-19 forecasting. For instance, in [6], a MOA model was developed that allows for the library of historic data to include data from 61 world regions. While incidence trends between regions can vary greatly in terms of noise and seasonality, the authors overcome this by first removing the noise and seasonality elements using the EpiInvert method of [7]. With normalized data across 61 different regions, the amount of historic data available was greatly increased, improving the performance of the MOA application to COVID-19 forecasting. In [8], a separate method was developed for comparing segments of COVID-19 incidence data across regions as a means to increase the amount of historical data available. In this method, a neural network is fit using data from many separate regions, and an attention mechanism is used to encode time series segments of separate regions in a form that isolates their comparable patterns. While both of these methods may be used to improve the practicality of MOA early on for forecasting an emerging epidemic, neither are capable of making reasonable forecasts until at least a moderate amount of data have already been observed. For the method in [6], forecasting only began after 150 days of COVID-19 count data were observed. The method in [8] uses data from the onset of the epidemic through the end of September 2020.

In this work, we develop a new, nonparametric method for disease forecasting in scenarios where there are little-to-no historical data. This method can be interpreted as both an extension of MOA that overcomes the requirement for a comprehensive library of historical data, and as a novel, competitive forecasting model for new, emerging epidemics. The key innovation of this method, called the *Synthetic Method of Analogues* (sMOA), replaces the library of related, historical time series with a rich library of synthetic time series generated to resemble a broad range of disease outbreaks. By replacing the unavailable historical time series library with an available synthetic time series library, sMOA circumvents the limitation of applying traditional MOA. Like MOA, sMOA does not need to estimate parameters concerning the behavior of a disease, such as disease transmission and recovery rates, to generate a forecast. Rather, a distance metric to compare the observed time series to the library of time series (or observed time series segment to library of time series segments) is all that is required.

Alongside sMOA, we develop a novel method for uncertainty quantification (UQ) based on online updates to the negative binomial regression model (MOA in [2] did not provide a method for producing forecast uncertainty intervals). While we demonstrate the performance of this UQ method using sMOA, the proposed UQ methodology can be applied to any model or method that generates point forecasts for count data that are well-represented by a negative binomial distribution.

This paper is organized as follows: Sect 2 outlines sMOA as an extension of MOA that uses synthetic data. We then describe the synthetic data generation procedures and how they are combined to produce forecasts. Additionally, we present a novel UQ method for nonparameteric procedures. In Sect 3, we compare the performance of sMOA with several models from the U.S. COVID-19 ForecastHub [9,10] in forecasting the early stages of the COVID-19 pandemic in 2020. In Sect 4, we discuss our findings and explore the future implications of this method.

## 2. Methods

In this section, we provide an overview of sMOA (Sect 2.1), a description of how the synthetic disease outbreak data are generated (Sect 2.2), describe how the sMOA hyperparameters are selected (Sect 2.3), and detail how uncertainty intervals are added to point forecasts (Sect 2.4).

### 2.1. Synthetic method of analogues

**Fig 1:**
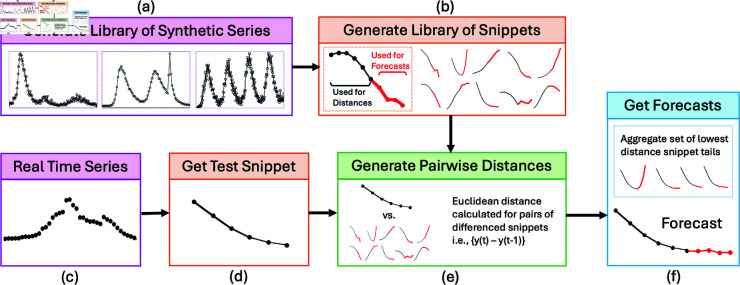
Diagram of sMOA. Recall *k* is the time series segment length and *h* is the largest forecast horizon. (a) Three fully observed synthetic time series ${\tilde{y}}_{i}^{ℒ}$ in the library. (b) Synthetic time series segments $y_{i}^{ℒ}$ of length k+h. The first *k* time points in black; the last *h* time points in red. (c) Fully observed time series ${\tilde{y}}^{𝒪}$. (d) Time series segment $y^{𝒪}$ of length *k* (i.e., the last *k* observations from the time series in (c)). (e) Compute the distance $d_{i}=d(y^{𝒪},y_{i,1:k}^{ℒ})$ between the observed time series segment $y^{𝒪}$ and the first *k* observations of each synthetic time series segment $y_{i}^{ℒ}$ in the library (i.e., the black points). (f) The point forecast is an aggregation (e.g., average) of the last *h* observations of the synthetic time series (i.e., the red points) with the smallest distances *d*_*i*_.

sMOA follows a procedure similar to the original MOA for disease forecasting from [2]. Fig 1 provides a diagram of sMOA (which is roughly the same diagram for the MOA method if “synthetic" is replaced with “historical"). First we provide some notation and connect it to Fig 1 for clarity. Let

- ${\tilde{y}}^{𝒪}$ be the observed time series of length *T* (e.g., Fig 1 (c)).
- *k* be the length of a time series *segment*. *k* is a hyperparameter chosen by the user. *k* must be less than or equal to *T*.
- $y^{𝒪}$ be the last *k* observations of ${\tilde{y}}^{𝒪}$ (i.e., $y^{𝒪}\equiv {\tilde{y}}_{(T-k+1):T}^{𝒪}$) (e.g., Fig 1 (d))
- $\tilde{N}$ and *N* be the number of synthetic time series and synthetic time series *segments* in the library, respectively.
- ${\tilde{y}}_{l}^{ℒ}$ be the $l^{\text{th}}$ synthetic time series in the library of length *T*_*l*_, for $l\in 1,2,\ldots ,\tilde{N}$ (see Fig 1 (a) for examples).
- *h* be the largest forecast horizon (chosen by the user, but often dictated by the forecasting objective).
- $y_{i}^{ℒ}\equiv y_{l,j}^{ℒ}$ be the $j^{\text{th}}$ time series segment of time series *l* of length k+h for $j=1,2,\ldots ,T_{l}-(k+h)+1$. For example, $y_{1,1}^{ℒ}$ are the first *k* + *h* observations of synthetic time series *l* = 1. See Fig 1 (b) for examples. As the main computational unit in sMOA is a time series segment (not the full time series), we drop the *l*,*j* indices and just use *i*.
- $d_{i}=d(y^{𝒪},y_{i,1:k}^{ℒ})$ be the distance between the observed time series segment $y^{𝒪}$ and the first *k* values of the synthetic time series segment $y_{i}^{ℒ}$. For now, *d*() is a generic distance function measuring how close the observed and synthetic time series segments are (see Fig 1 (e)).
- ${𝕐}_{m}^{ℒ}$ be the *m* synthetic time series segments with the smallest distances *d*_*i*_, where *m* is a hyperparameter chosen by the user.
- $a({𝕐}_{m}^{ℒ})$ be a generic aggregation function (e.g., average or median) that summarizes the *m* synthetic time series segments in ${𝕐}_{m}^{ℒ}$ into a point forecast for each forecast horizon between 1 and *h* (see Fig 1 (f)).

In this paper, we use the convention that $a^{“\sim ”}$ represents full length time series and the absence ${\mathrm{of}}^{“\sim ”}$ represents time series segments.

With notation defined, MOA and sMOA can be described. For a given time series segment of length *k*, a max forecast horizon *h*, a library of historical/synthetic time series segments each of length k+h, a distance function *d*(), and an aggregation function *a*(), the method for producing a point forecast proceeds as follows:

1. Compute distance *d*_*i*_ for all *N* time series segments in the library.
2. Identify the *m* time series segments in the library with the smallest distances. Collectively call them ${𝕐}_{m}^{ℒ}$.
3. Define the point forecast for steps-ahead 1 through *h* as an aggregation of the nearest time series segments in the library: ${\hat{y}}^{F}=a({𝕐}_{m}^{ℒ})$.

sMOA can be broadly understood as MOA with a synthetic data library replacing the historical data library. However, several implementation choices distinguish sMOA from MOA, many of which are also essential for applying the original MOA. These implementation choices (sometimes called hyperparameters) include:

- *k*: the length of the time series segment
- *N*: the number of historical/synthetic time series segments to include in the library
- *d*(): the metric measuring the distance between the observed time series segment and each historical/synthetic time series segment
- *m*: the number of closest historical/synthetic time series segments to use in the aggregation calculation
- *a*(): the aggregation function that takes as input the *m* closest time series segments from the historic/synthetic library and outputs a point forecast for horizons 1 through *h*. Examples of *a*() include computing an average or median.

Selecting these implementation choices is not arbitrary and requires care. [2] used a cross-validation approach to make these choices for the MOA. For the sMOA instance developed in this paper, the hyperparameters were chosen according to a novel Bayesian optimization scheme (as described in Sect 2.3).

### 2.2. Synthetic data generation

The underlying philosophy of sMOA is that a synthetic library representing a rich and diverse collection of *possible* disease outbreaks will function in a way that is comparable to MOA with real historical data. While we don’t know how an emerging outbreak will unfold, we do have historical outbreaks for a variety of diseases to lean on. Generating synthetic disease outbreak data requires a few ingredients: (1) a parameterized forward model to generate synthetic outbreaks and (2) ranges of parameter values that conservatively “cover" historical values.

For the presentation of sMOA in this paper, the synthetic outbreak data are primarily generated from a Susceptible-Infectious-Recovered (SIR) compartmental model [11]. In the SIR model, a population is divided into compartments that describe each individual’s current health state; individuals in these compartments change states based on a deterministic system of ordinary differential equations. Denote the proportion of the population in the susceptible, infectious, and recovered compartments by $S_{t},I_{t},$ and *R*_*t*_, respectively, such that $S_{t}+I_{t}+R_{t}=1$ for all *t*. Then the SIR model is determined by the equations

$$\frac{dS_{t}}{dt}=-\beta S_{t}I_{t},$$ (1a)

$$\frac{dI_{t}}{dt}=\beta S_{t}I_{t}-\gamma I_{t},$$ (1b)

$$\frac{dR_{t}}{dt}=\gamma I_{t},$$ (1c)

where β>0 is the disease transmission rate and γ>0 is the rate of recovery. If one knew these two rates, and the initial number of individuals in each category – $S_{0},I_{0}$ and *R*_0_ – the numbers $S_{t},I_{t},\text{and}\;R_{t}$ could be numerically simulated for any time-point *t* using the above system of differential equations.

The basic reproduction number, $\rho :=S_{0}\beta /\gamma$, is a common summary metric of an SIR curve (this is often assumed to approximately be equal to β/γ as *S*_0_ is allowed to approach 1); it represents the expected number of new infections of a disease generated by one infectious agent in a completely susceptible population. The basic reproduction numbers of the SIR curves used to generate the synthetic data range from ~1.1 to ~19.2. This is a broad range of basic reproduction numbers that covers the estimated ρ’s for several diseases, including COVID-19 [12], influenza [13], monkeypox [14], Dengue fever, Zika, and Chikungunya [15].

One short-coming of an SIR model is that it can generate at most a single outbreak or wave. Real outbreaks, however, can be composed of multiple waves. We stitch together multiple SIR outbreaks (each initialized with a different set of parameters/initial conditions) by randomly offsetting their start times (see Fig 1 (a) for examples of multi-wave outbreaks generated in this way). Additional modifications are made, such as mapping these SIR curves to counts or proportions to cover a wide variety of possible scales, and adding noise to the data; those details are discussed in S1 Text, and visualized in Fig A in S1 Text.

Before continuing, two points need to be addressed. First, it’s worth stating explicitly that the goal of the synthetic outbreak generation is not to adequately predict the anticipated course of the outbreak and synthetically generate as many possible outbreaks “near" that prediction. Rather, the synthetic library should be thought of as a wide net covering many possible disease outbreaks. Because the library is synthetic, we can generate as many outbreaks as are needed. So long as the true and yet-to-be-observed outbreak segment is contained within the range of synthetic outbreaks, we believe sMOA can be successful. It’s possible/likely many of the synthetic time series segments are not close to the observed time series segment. This should not impact the forecast, however, as these far away synthetic segments will not be part of the closest *m* time series segments and thus won’t be used in the aggregation function producing the forecast.

Second, the underlying assumption of sMOA is that time series segments from real outbreaks can be *locally well-approximated* by segments of SIR model outputs (or a summary statistic of several such outputs). The assumption that an outbreak can be locally approximated by a simple compartmental model has precedent [16–18]. Typically, this assumption manifests itself by replacing static parameters of compartmental models with time-varying ones. In sMOA, “time-varying parameters" are being functionally replaced by “time series *segments*."

### 2.3. Bayesian optimization for hyperparameter selection

Within sMOA, there are several hyperparameters and modeling choices that must be made *a priori*. The hyperparameters that must be selected were listed in Sect 2.1 but are restated here: time series segment length *k*, number of time series segments included in the library *N*, distance metric *d*(), number of closest time series segments *m*, and aggregation function *a*(). Experiments on real and synthetic data show that these hyperparameters all have a strong effect on the performance of sMOA. Furthermore, there is not a clear context for these values that might help a researcher determine appropriate values for these parameters. In general, these facts would make any future application of sMOA challenging, since sMOA is intended for scenarios where a disease has not previously been observed, and thus there would not be any existing intuition for a subject matter expert to use in selecting these hyperparameters. To address this issue, we develop an automated process for selecting these hyperparameters using Bayesian optimization that operates exclusively on synthetic data.

We start with separate synthetic data set than the one within sMOA: the synthetic test set. This separate data set was generated following the procedure of Sect 2.2. Our goal is to find the hyperparameter inputs (specifically, *N*, *k*, and *m*) that minimize the forecast error when applied to the synthetic test set. Here we measure forecast error on the synthetic test set with mean absolute error (MAE). In our set up, the synthetic test set is being used as a proxy for real data (which, again, would not be available at the time of hyperparameter determination in an emerging disease setting). Finding the hyperparameters that minimize the synthetic test set forecast error is an optimization problem.

We’ve chosen to use Bayesian optimization, a global optimization procedure [19], to perform our optimization task. Bayesian optimization has three steps: (1) an initial exploration step, (2) an emulator fitting step, and (3) a sequential search step. Bayesian optimization begins by drawing a Latin hypercube sample [20] on the input space (*N*, *k*, and *m*) and evaluating the forecast error of sMOA under those hyperparameter choices. Thus, the result of the initialization step is a collection of input/output pairs, where the input is the sampled (*N*,*k*,*m*)-tuple and the output is the scalar MAE over the synthetic test set. Call this collection of input/output pairs the *emulator training data*. The second step requires learning a function that maps inputs (hyperparameters) to the output (MAE) based on the emulator training data. In the Bayesian optimization literature, the model used to estimate the function mapping inputs to output is most commonly a Gaussian process. Thus, the emulator is a fitted Gaussian process. With an emulator in hand, we use it to find the input parameter that minimizes the output (i.e., the hyperparameter setting that is predicted to minimize MAE over the synthetic test set). Call this input the *candidate minimizer*. We then compute the actual MAE over the synthetic test set for the candidate minimizer, add the candidate minimizer and its corresponding MAE to the emulator training data set, and refit the emulator to the updated emulator training data set. This process repeats for a pre-determined length of time or for a pre-specified number of iterations. For the application in this paper, we ran this process for 5 iterations. At the end of this algorithm, the hyperparameters are set to the candidate minimizer. The full code to perform this process is available on this paper’s Github page (https://github.com/lanl/precog/tree/main/smoa).

The process described above determined an *N* of 18,387, a *k* of 5, and an *m* of 4,422; these were all used in the application for this paper. The distance metric *d*() is taken to be the summed, point-wise absolute differences between the time series segments. Working with first-order differences of time series segments rather than the segments themselves addressed the different scales of synthetic data. For the aggregation function *a*(), we selected the median of the *m* closest time series segments, rather than the average. The authors believe that the median produces a better point forecast because it protects against instances of high outliers and sporadic behavior in the synthetic data library. This decision was also motivated by the study in [6], which found that the median had slightly better performance than a weighted average for MOA. In future work, we would explore Bayesian optimization for both continuous and categorical inputs.

### 2.4. Uncertainty quantification (UQ)

The method described in Sect 2.1 only produces a point forecast for a given forecast horizon without incorporating any UQ. The authors explored several possible methods to get UQ for sMOA. One approach under consideration was to use the quantiles of the closest synthetic time series in ${𝕐}_{m}^{ℒ}$. While this may give reasonable prediction intervals for a forecast, it presents a conceptual contradiction. As discussed in Sect 2.2, the aim in creating the synthetic data library is to capture a wide range of variability. However, increasing variability in the synthetic library would likely lead to an *a priori* inflation of the range in the quantiles of ${𝕐}_{m}^{ℒ}$ irrespective of the behavior of the actual observed data.

To avoid the potentially conflicting aims of maximizing variability in the synthetic library while also getting reasonable UQ, we develop the following UQ method based on the negative binomial regression (NBR) model [21]. The NBR model is appropriate for this application because the data consists primarily of non-negative counts, and for which variance of the observed data is typically greater than the mean [22]. The probability density function of the NBR model is given by,


$$ℒ(Y=y)=\frac{\Gamma (r+y)}{y!\Gamma (r)}{\left(\frac{r}{r+\mu }\right)}^{r}{\left(\frac{\mu }{r+\mu }\right)}^{y},$$

where *y* is the number of counts observed, μ is the mean, and *r* is the *dispersion term* that controls the overall variance of the model. Specifically, E(Y)=μ and $\text{Var}(Y)=\mu +\frac{\mu^{2}}{r}$.

The UQ method developed here fits an NBR model with a mean μ equal to the point forecast produced by sMOA. For the dispersion term *r*, *h* separate estimates are made (one for each forecast horizon) using the recent history of data observations for *y*. The motivation for individual estimates for every horizon is because we expect the certainty of estimates to go down (and therefore the dispersion term to become smaller) for forecasts further out in time. With an estimation for *r* and μ for a given forecast horizon, prediction intervals can be calculated by integrating *y*. A notable strength of this set-up is that it uses both synthetic data and recent-history data to quantify uncertainty in data-limited scenarios, and thus it is applicable to emerging diseases.

The dispersion term for the NBR model is fit using a Maximum Likelihood Estimator (MLE) on the recent history of forecasting errors, assuming that there are around 11 weeks of data. Once there are 11 such observations $\left\{y_{1},\ldots ,y_{11}\right\}$, there would then also be 11–*k* one-step-ahead forecasts from sMOA $\left\{f_{k+1},\ldots ,f_{11}\right\}$ for which the forecasting errors are explicitly known. Similarly, there would be 10–*k* such observations for all two-step ahead forecasts, 9–*k* such observations for three-step ahead forecasts, and so on. Note that this assumes 11–*k*–*h*>0. Using these newly-acquired observations, the likelihood


$$ℒ(\{y_{k+1},\ldots ,y_{11}\})=\prod_{i=k+1}^{11}\frac{\Gamma (r+y_{i})}{\left(y_{i})!\Gamma (r\right)}{\left(\frac{r}{r+f_{i}}\right)}^{r}{\left(\frac{f_{i}}{r+f_{i}}\right)}^{y_{i}}$$

can be maximized with respect to *r*; this maximum for *r* is then used as the dispersion term for the *next* one-step-ahead forecast. This calculation is very fast via the optim function in R [23], and is easily repeated for each forecast horizon, and for each new data point that is observed. Note that this setup assumes that the forecasting errors are independent.

The choice of the 11-week cutoff is to ensure that there are enough data to reasonably fit an MLE; dispersion terms prior to this span of time must either be selected by the researcher or determined according to a synthetic Bayesian optimization scheme (such as in Sect 2.3). Initial experimentation with this method showed that having too few observations at the onset of a disease outbreak led to MLE estimates of *r* close to zero. Since a dispersion term below 1 places the mode of the NBR model at zero, the constraint of *r* > 1 was added to this procedure.

## 3. Application to the COVID-19.ForecastHub

### 3.1. Demonstration of sMOA early pandemic forecasts

While sMOA is intended to forecast *emerging* epidemics for which there is little-to-no historical data, we will show that it has competitive performance during all stages of an epidemic. To evaluate this performance, we apply it to early COVID-19 case data available through the COVID-19 ForecastHub. The ForecastHub is a central repository for data and forecasts on COVID-19 that first began in March 2020 at the University of Massachusetts Amherst. Predictions on the forecasted number of new cases, hospitalizations, and deaths in the US for future days, weeks, and months were submitted by teams across the world [10].

**Fig 2:**
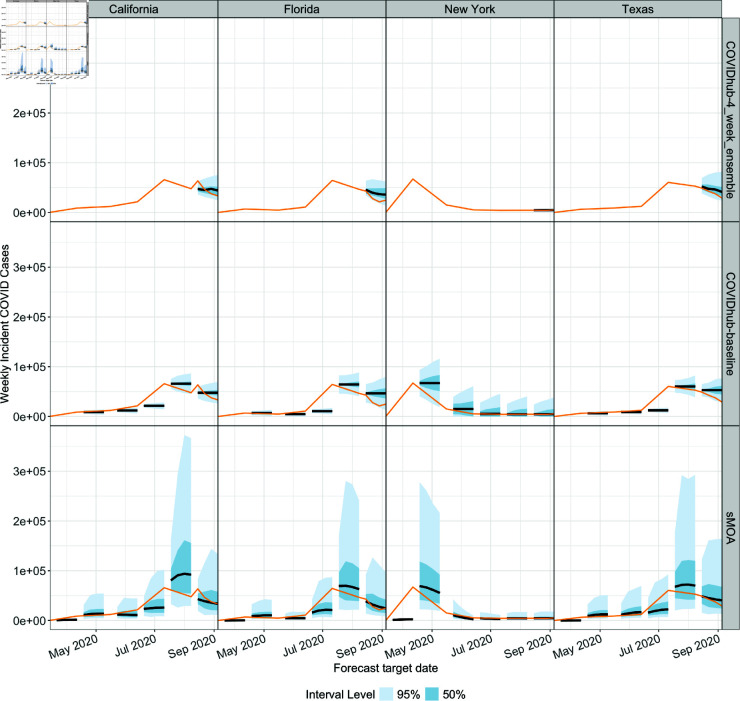
A demonstration of sMOA forecasting during the early weeks of the COVID-19 epidemic. Black lines correspond to point forecasts; the orange lines correspond to the true observed value. The basic ensemble model of the ForecastHub (‘COVIDhub-4_week_ensemble’) and the basic persistence model (‘COVIDhub-baseline’) forecasts are provided for reference for the dates where forecasts were provided. The third model used for later comparisons, the ‘COVIDHub-trained_ensemble’, does not provide forecasts this early in the COVID-19 epidemic.

As an initial demonstration, we calculate the sMOA forecast for the earliest available dates data in the ForecastHub for the four most populous US States in Fig 2. The four most populous states were chosen because they represented the most people of any set of four examples in the US. Note that although sMOA has relatively wide prediction intervals, it often captures the behavior of the true data. While we also provide the ForecastHub ensemble model (‘COVIDhub-4_week_ensemble’) and the persistence model (‘COVIDhub-baseline’) as references for reasonable forecasts, these forecasts are not directly comparable to the forecasts given by sMOA for two reasons. First, these two models do not provide forecasts as early as sMOA is able to provide forecasts. Second, since data for a given date are corrected as more information becomes available later in the pandemic, the data available now may not be the same used by the ForecastHub modelers at the time they were creating their forecasts. The ForecastHub provides two versions of data for a given date – an “as-of" value that reflects how the data looked at the time, and the “final" data that is the most accurately known truth for that date. Since this “as-of" feature is only available for dates starting August 2020, these early forecasts from sMOA are necessarily calculated using a slightly altered dataset than the two reference models.

**Fig 3:**
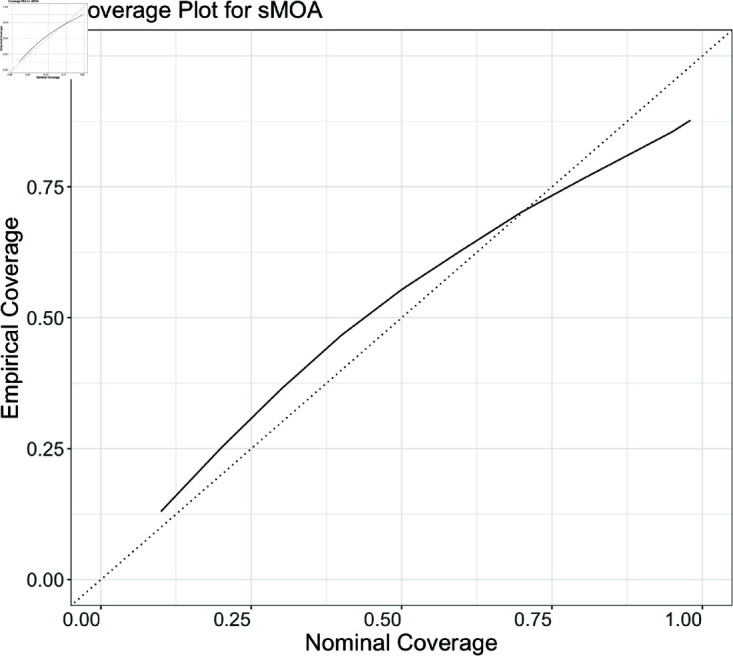
Nominal vs. empirical coverage for sMOA over every state in the US and over four forecast horizons (1w, 2w, 3w, 4w), plotted using a black line. The dotted line indicates a perfect match between nominal and empirical coverages for reference. Over every forecast made for the data application to COVID-19, nominal and empirical coverages approximately match.

The prediction intervals for sMOA are wider than the two reference examples in Fig 2 because the negative binomial model can be very skewed, especially in cases of low certainty. However, it is possible that these wide prediction intervals may be an improvement over the intervals in the ForecastHub, as it was found that a majority of COVID-19 case forecasting models in the ForecastHub exhibited (occasionally dramatic) undercoverage at the 50%, 80% and 95% levels (see [24], Fig 3). In the following section, we investigate these wide prediction intervals across the entire pandemic.

### 3.2. Coverage analysis of sMOA forecasts

We investigate sMOA’s predictive coverage in Fig 3. For this analysis, we use all available disease data from the ForecastHub for COVID-19 in the U.S. following August 2020, thereby only using data for which an “as-of" date is available. A coverage calculation investigates the proportion of time a given prediction interval captures the true value on a hold-out set of data. For instance, a well-performing prediction interval at the 95% level should capture the true value approximately 95% of the time [25].

For every date, horizon, and state for which a sMOA forecast is calculated, we record the coverage of the central 10%, 20%, 30%, 40%, 50%, 60%, 70%, 80%, 90%, 95% and 99% probability regions. Fig 3 compares the expected (Nominal) coverage to observed (Empirical) coverage. Good agreement can be observed. Based on Fig 3, there is slight evidence of overcoverage at 10-70% regions, with some evidence of undercoverage for the 80% and higher regions. Based on this result, we believe that sMOA tends to have high uncertainty at the onset of a pandemic, which is appropriate since this is the period of highest uncertainty. To investigate this further, we visualize a collection of forecasts later on in the pandemic in Fig C in S1 Text.

While the forecast interval widths appear more defensible later on in the pandemic, the high uncertainty presents real challenges for communicating forecasts and using them to make decisions. This being said, the large prediction interval widths may nonetheless be appropriate. For instance, while the 4-week ahead, sMOA forecast in late July 2020 in California reaches above 350,000 (Fig 2), the highest reported number of cases in California in a single week was well above that (852,280) during the Omicron wave in late 2021/early 2022.

### 3.3. Comparison of sMOA to ForecastHub models

We consider the task of forecasting reported COVID-19 incident cases between the months of August 2020 and March 2023. We focus on one- through four-week out forecasts (*h* = 1w, 2w, 3w, 4w) at the state level in the US; excluding any US territories. Using the covidHubUtils package in R [26], the real-time data available to all models in the ForecastHub *for any historical date* after mid-August 2020 can be easily acquired. By restricting sMOA to the data available to the ForecastHub models for a given date, a direct comparison can be made between sMOA and each individual model in the ForecastHub. Note that the ForecastHub did not begin recording the “as-of" until mid-August 2020 (data available in the covidHubUtils package prior to this date has been corrected for errors, and is not the same data that would have been available to scientists at the time), which is why this portion of the study focuses on data after mid-August 2020. Since the ForecastHub is a repository for forecasts submitted by independent teams, forecasts for all dates are not consistently available for every model. Consequently, direct comparisons with individual models in the following sections are limited to dates, horizons, and states where forecasts are available for that model. We include all models that submitted at least 11 cases forecasts between the August 2020 and March 2023. Out of 61 models on the ForecastHub, 57 models met this criterion for point forecasts and 54 met it for quantile forecasts.

While we compare sMOA to the models in the ForecastHub as a way to demonstrate its performance during an emerging epidemic, the intent of this study is not to claim that sMOA is better than any of the models that submitted forecasts to the COVID-19 ForecastHub. sMOA benefits from both an ability to retroactively evaluate performance during development (although at no point was sMOA exposed to real data during training, the authors were aware of the model’s accuracy on actual data throughout its development), and from an abundance of time for research when compared to the time pressures of developing these models in the midst of a pandemic. Rather, the intent of this study is to show that sMOA has competitive performance despite relying on synthetic data for its forecast. The UQ method requires no data initially but incorporates observations as they become available, and no explicit parameter estimation is required (outside of the negative binomial’s dispersion parameter). As we discuss later, developing a method that allows for the mean forecast to incorporate observations as they become available is a direction for future research. Calculating forecasts with this model is also computationally fast; given that the synthetic data are pre-built, all forecasts calculated for this analysis together took ~15 minutes when run in parallel on a Linux environment. Over all states, dates, and forecast horizons, this was a total of 23,068 forecasts, using 50 CPUs. Thus, sMOA is well-suited for instances where historical data are not available and disease dynamics are evolving in real time, making it viable for future emerging disease outbreaks.

In the following, we consider two error measures to assess the merit of the forecasts produced by sMOA. These error measures are Mean Absolute Error (MAE) and Weighted Interval Score (WIS). Each of these measures are common in the disease forecasting literature [27–29]. The WIS is a score that assesses predictive accuracy across multiple confidence levels (see [30]). The WIS thus summarizes more information than the MAE, which only assesses a point forecast. The confidence levels used for the WIS calculations in this paper are the same as those required for submission to the ForecastHub (7 quantiles); the weights for the average used to calculate WIS were also the same as the ForecastHub [10].

We compare sMOA against the models that submitted to the COVID-19 ForecastHub under the criterion discussed above. As mentioned previously, all direct comparisons to individual models are limited to dates, horizons, and states where forecasts are available. For instance, when the mean MAE is calculated for the “COVIDhub-4_week_ensemble" model, it is compared to sMOA’s mean MAE, which is based on forecasts only from the same dates, states, and horizons where “COVIDhub-4_week_ensemble" forecasts are available.

**Fig 4:**
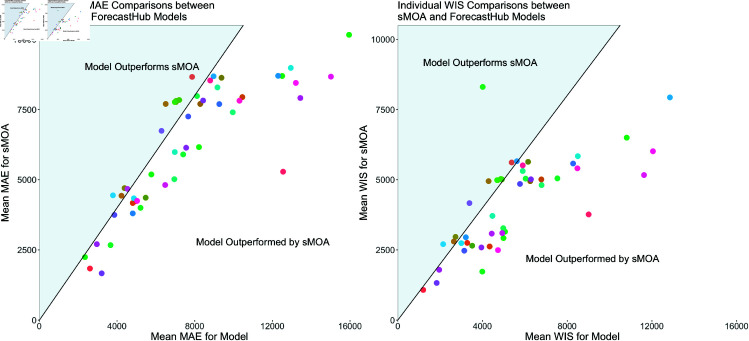
Direct comparisons between models from the ForecastHub and sMOA, using mean MAE (left) and mean WIS (right). The error comparison between sMOA and a given model from the ForecastHub is only calculated for the dates for which forecasts from the given model were reported. That is, a given point represents the mean error metric for a model from the ForecastHub calculated over every date, state, and forecast horizon available for that model, plotted against the same mean metric calculated using sMOA on these same dates, states, and forecast horizons. Models beneath the diagonal black line were outperformed by sMOA. Four outlier models were removed for ease of visualization.

Direct comparisons between sMOA and the individual models in the COVID-19 ForecastHub are shown in Fig 4. The first observation is that sMOA – without using any historical data – has a better performance than a majority of models both in terms of MAE (out-performing ~78% of models) and in terms of WIS (out-performing ~76% of models). Four outlier models, one of which out-performed sMOA while the other three did not, were removed from this plot to make the plot more readable.

Unlike many of the models in Fig 4, sMOA does not rely on demographic or mobility data to generate forecasts, nor does it make any explicit assumptions about social distancing or other behavioral changes over the prediction period. Additionally, it requires no explicit parameter estimation for factors such as disease transmission and recovery rates. Even though sMOA does not use historical data, it is able to out-perform a majority of the models in the ForecastHub both in terms of average MAE and average WIS.

### 3.4. Contextualizing sMOA results

sMOA, which relies solely on synthetic time series, outperforms 76% - 78% of the models submitted to the ForecastHub. While these results are promising, this finding warrants further investigation. For instance, it is worth asking if sMOA is outperformed by “best-in-class" models. To investigate this question, we partitioned all ForecastHub models into 10 “best-in-class" models and the remaining “core" models. The 10 best-in-class models were chosen as the 10 models that had WIS scores comparable to or better than the COVIDhub-baseline model between July 2020 and December 2021 across all states and territories, as defined in Fig 2(b) of [24].

**Fig 5:**
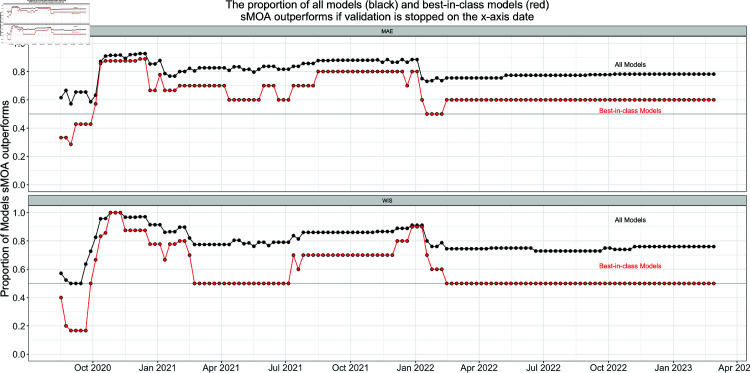
The proportion of all models (black) and best-in-class models (red) sMOA outperforms in MAE (top) and WIS (bottom) if the validation window ranged from August 2020 through the x-axis date. sMOA outperforms the majority of all models and best-in-class models if the validation date cut off is between October 2020 and March 2023. Directly before October 2020, there was a dip in incidence case counts that sMOA failed to forecast accurately that caused the initial lower performance.

Fig 5 shows the proportion of all models and best-in-class models sMOA outperforms if the validation window ran from August 2020 through the x-axis date. The results in Sect 3 correspond to the last date in Fig 5. While it is true that sMOA performs better when compared to all ForecastHub models than it does when compared to just the best-in-class models, sMOA does outperforms at least half of each set of models for both MAE and WIS. Furthermore, this result is not a product of a carefully chosen validation window. If the validation window end date were any date after October 2020, sMOA would have outperformed at least half of all ForecastHub models and best-in-class models in both MAE and WIS. This is strong evidence that sMOA is not just beating core models, but also best-in-class models.

## 4. Discussion

sMOA has a strong potential for rapid application in public health contexts where data limitations are common, such as the case of an emerging epidemic, whenever the disease is represented by the synthetic library. By creating a comprehensive library of synthetic data *a priori* that represents many possible behaviors of an infectious disease outbreak, sMOA addresses several challenges inherent in forecasting during the early stages of an emerging outbreak. A key challenge is the lack of historical data on the behavior of a previously unobserved disease, which limits the utility of traditional models – like the original MOA – that rely on matching historical trends to current disease behavior. Models that require the estimation of parameters such as disease transmission and recovery rates will also suffer from a lack of historical data. The synthetic data mechanism within sMOA circumvents the need to estimate model parameters by instead simulating several possible disease dynamics and matching these dynamics to data observations once the data become available. Since the synthetic data are simulated *a priori*, this matching process is computationally fast and produces rapid predictions.

We have shown in this paper that sMOA has competitive forecasting potential compared to the models in the COVID-19 ForecastHub. Since sMOA was only given the historical data that were available at the time of the forecast date, the model forecasts are comparable to a model that was producing forecasts in the midst of the pandemic. This study is evidence that, at least in the case of the emerging COVID-19 epidemic, sMOA is able to provide competitive forecasts on infectious disease trends with little-to-no historical data. The excellent performance of sMOA is encouraging, especially since – unlike many of the models from the ForecastHub – sMOA did not use any data or assumptions regarding behavioral patterns or public health mandates implemented during the pandemic. For this reason, we hypothesize that future applications of sMOA will be robust against major changes in a disease landscape, such as forecasting during and after intervention efforts. Future studies on sMOA will investigate its forecasting potential on such disease scenarios as well as its applicability to other historical disease outbreaks. As a means to motivate the exciting potential for future research in this direction, we provide some initial analyses on applying sMOA to other infectious diseases in the Supplementary Materials (in S1 Text and visualized in Figs D & E in S1 Text). We take four additional disease datasets – influenza hospitalizations, ILI incidence, Dengue fever, and Chikungunya – and compare the sMOA forecasts against the basic persistence model forecasts. We also analyze what observed disease dynamics are and are not covered by the synthetic data library (Fig F in the S1 Text).

There are several additional directions for future work. The synthetic data library developed for this paper was *ad hoc* and there is ample room for further experimentation and development. In general, enhancing the synthetic data generation process is expected to lead to more accurate forecasts. In this vein, there are many directions for future work. We could

- augment/replace the synthetic data library with a library of all available infectious disease data.
- fold in real data from the emerging disease as it becomes available.
- enhance the biological realism of our synthetic generation processes through the use of agent-based models [31–33] that naturally incorporate seasonal and evolutionary mechanisms for multiple case waves [34].

That is, replacing a synthetic library with an *available* library composed of both historical data and synthetic data from a variety of generative mechanisms may represent the best of both worlds and, importantly, would be accessible in any emerging disease forecasting context.

Another direction for future work is at the Bayesian optimization stage. In this paper, we only used Bayesian optimization to select the continuous parameters of sMOA: *N*, *k*, and *m*. Extending Bayesian optimization into a mixed data type regime, where the discrete choices of distance metric *d*() and aggregation function *a*() would augment the generalizability of this work [35].

We only explored sMOA as a standalone forecasting model. Because sMOA can quickly generate forecasts, it could easily be treated as a component model in an ensemble.

While the UQ method developed in this paper was competitive when compared to the models in the COVID-19 ForecastHub, there is potential for further development. Future work will investigate whether the same approach used to produce a mean forecast can be extended for variance forecasting. This may improve the accuracy of the prediction intervals produced by the UQ method. With these improvements, sMOA could become an even more powerful tool for epidemic forecasting in a wide range of outbreak scenarios, including novel and evolving disease landscapes.
