# Correction: Synthetic method of analogues for emerging infectious disease forecasting

**DOI:** 10.1371/journal.pcbi.1013627

**Published:** 2025-10-30

**Authors:** The PLOS Computational Biology Staff

S1 Text is omitted from the list of Supporting Information. It can be viewed below.

## Supporting information

S1 Text (PDF)

The publisher apologizes for the error.
