# Supplementary material for: Correction: Synthetic method of analogues for emerging infectious disease forecasting
Source: PLoS Comput Biol. 2025 Oct 30;21(10):e1013627. doi: 10.1371/journal.pcbi.1013627 (PMC12574889; doi:10.1371/journal.pcbi.1013627)
Supplement: S1 Text — (PDF) [file pcbi.1013627.s001.pdf]

S1 Text

S1.1 Synthetic Data Generation

In this section, we describe how the synthetic data library is created. For reference, we graph four examples of synthetic data created according to this process in Fig 1. Note that each time series contains several peaks, and the height and smoothness of these peaks can vary both within and between time series.

First, we describe some of our motivation behind our choices regarding synthetic data generation. The sMOA algorithm described in the main paper is based on 5-week segments of these synthetic time series, so long-term disease dynamics encoded into the synthetic data will not impact forecasts; rather, the forecasts will be totally driven by local synthetic data dynamics. An advantage of this local property of our sMOA forecaster is that our synthetic time series realizations do not need to approximate an entire real data time series; instead, the goal is that local sections of the synthetic time series could potentially match local dynamics observed in real data.

In order to capture temporally-local disease dynamics, we return to a simple and fundamental model common in infectious disease modeling: the susceptible-infected-recovered (SIR) model. On its own, this model is often inadequate for modeling complicated long-term disease dynamics, but we argue that this model can provide the building blocks necessary to capture short term disease dynamics. In the short term, we are mostly interested in capturing cases attributed to a single case wave or nearly-co-temporal case waves related to distinct disease variants/subtypes.

With these principles in mind, we propose generating our synthetic data as *mixture of SIR model incidence realizations*, where these combinations of SIR curves are randomly generated in an effort to fill (and ideally contain) a poorly-defined space of local disease dynamics we may possibly encounter in real data across a variety of diseases. We consider three variations of these SIR model mixtures: (1) a "rollercoaster" model, where SIR realizations are randomly stitched together along the time axis, (2) a "wiggle" variation on the rollercoaster model, where periodic oscillations/deviations from a standard SIR model are added, and (3) a "seasonal" model, where consecutive SIR realizations either follow the same incidence curve or a similar incidence curve with a temporal trend. All variations have additional noise added to better approximate real data observations. While we can imagine many alternative strategies for generating simulated data to augment this set (e.g., simulations with occasional outliers added), we view this set of simulated data as a good starting point for capturing relevant temporally-local dynamics for COVID-19 and other infectious diseases.

All three of our SIR mixture variations (detailed below) are based on combining multiple SIR "waves" – periods of time where the observations (either counts or proportions) see a rapid increase followed by a rapid decrease, generated via the SIR compartmental model and then combined in distinct ways

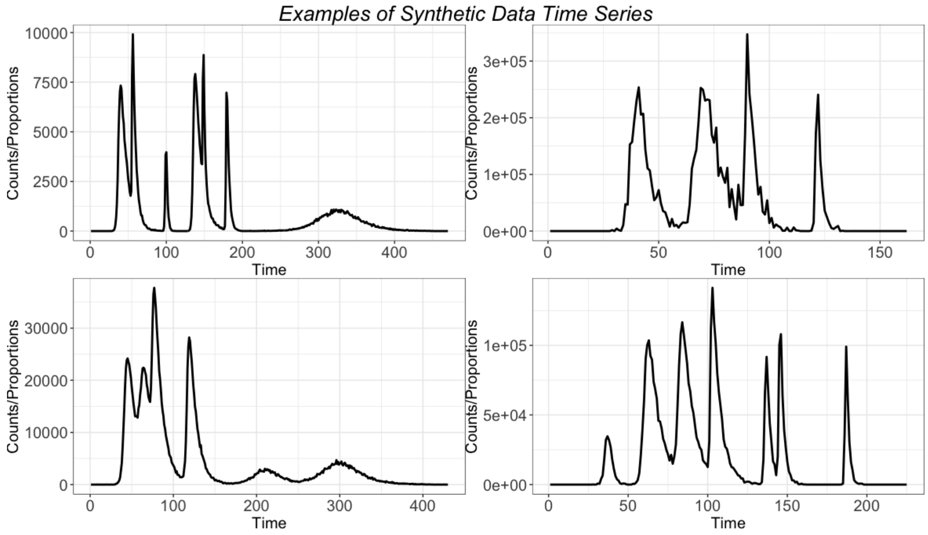

Fig A: Four examples of synthetic data time series in the synthetic library.

to produce a single datum (time series) in the synthetic library. We begin by covering the methods by which these waves are generated; following this, we will cover the methods by which these waves are combined into an entire synthetic time series, including the three variations mentioned above. At the end of this supplement, we analyze the coverage of the synthetic data library on several known diseases. Note that the term "coverage" in the supplementary material is not being used in a probabilistic forecasting sense, but rather in the sense of spanning or covering. We are investigating whether or not the synthetic time series segments resemble the time series segments of real outbreaks. We further perform forecasting on all of these known diseases using sMOA and compare its performance against a baseline persistence model.

The procedures described below produce $\sim 60,000$ full time series ($\sim 20,000$ from three distinct procedures). Note that within the sMOA model, these synthetic data are split into every possible segment of length $k + h$; the set of all possible $k + h$ segments is the synthetic data that are used to calculate a sMOA prediction. This "look-up step" – where an observed time series of length k is compared against every segment of length k in the synthetic data library – is performed using the segments in this set. Transforming the full synthetic time series into segments resulted in 15,642,758 different possible segments in the synthetic data library; these are the segments used for the applications in this paper.

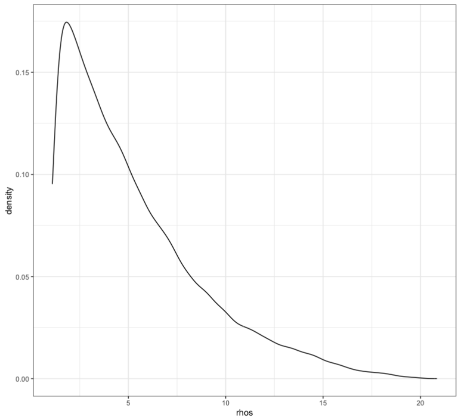

Fig B: Density of ρ values represented in the synthetic data.

Generating a Single Case Wave using the Susceptible-Infected-Recovered Model

A single "wave" is generated according to the infected category over time in an SIR model (from this point onward, we will refer to these values as an "SIR curve"). The SIR model, and the notation we will use in the following explanation, are introduced in the main paper ((1a)-(1c) and in the text before and after these equations). To generate a single SIR curve, the initial proportion of a population allocated to each compartment is sampled from

$$(S_0, I_0, R_0) \sim \text{Dirichlet}(1000, 0.1, 0.1),$$

while the starting parameters ρ and γ are sampled according to

$$\rho \sim 1.1 + \text{Beta}(1, 3) * 20; \qquad 1/\gamma \sim \text{Unif}(1, 10),$$

where $\rho := S_0\beta/\gamma$ is the basic reproduction number (this is often assumed to approximately be equal to β/γ as S_0 is allowed to approach 1). This sampling procedure gives ρ's approximately in the range of 1.1 to 19.2. The distribution of ρ values generated by this procedure is visualized in Fig B. This range of basic reproduction numbers covers the estimated ρ's for several diseases, including COVID-19 [1], Influenza [2], Monkey Pox [3], Dengue Fever, Zika, and Chikungunya [4]. For the time series created that are meant to mimic a seasonal trend (see Section S1.1.4 below), the ρ's were instead sampled from $1.1 + \text{Beta}(1, 3) * 10$.

S1.1.1 Combining Multiple Case Waves

Below, we describe three approaches we use to combine multiple SIR-based case waves. In all cases, we generate long synthetic time series combining dynamics from multiple SIR case waves. Since we are primarily interested in local case wave dynamics, an alternative approach would be to generate many more short

time series realizations. We chose to generate long time series for data storage convenience.

S1.1.2 Susceptible-Infected-Recovered "Rollercoaster" Model

In this generative model, several Susceptible-Infected-Recovered (SIR) curves are sampled according to the process described above and combined in a way to simulate waves of infections in sequence. To construct each time series, a SIR curve is generated for each wave, where the number of waves is randomly sampled to be between 3 and 7 and the basic reproduction number and γ values are generated separately for each wave. These waves are placed along the time axis randomly, where the time between the start of consecutive waves is randomly drawn to be between 10 and 52 weeks. The SIR waves are ordered such that (a) waves with higher basic reproduction numbers are more likely to occur earlier on, (b) waves with higher basic reproduction numbers are more likely to occur later on, or (c) the ordering of the waves is not related to the basic reproduction number.

S1.1.3 Susceptible-Infected-Recovered "Wiggle" Model

In this generative model, an element of periodicity and deviation from SIR is added to the "rollarcoaster" framework from Section S1.1.2. Let $Z := \{Z_1, \ldots, Z_T\}$ be a synthetic time series sampled according to the process in Section S1.1. Each timepoint Z_t of Z is then rescaled,

$$Z'_t := Z_t \cdot [1 + (1 - 2A) \cdot (1 + B * \sin(\pi t C))],$$

where $A \sim \text{Unif}(1, 2), B \sim \text{Binomial}(0.5)$, and $C \sim \left(\text{Unif}\left(\sqrt{2/T}, \sqrt{10/T}\right)\right)^2$. The synthetic datum $Z' = \{Z'_1, \ldots, Z'_T\}$ is then added to the library.

S1.1.4 Seasonal Model

In this generative model, a single generated SIR curve is repeated seasonally, with a multiplicative trend over time. The number of repeated seasons is sampled randomly between 5 and 15, and the SIR curve is generated as described previously except that $\rho = 1.2 + 10X$ with X generated from a $Beta$ distribution as before. Seasons are defined on a 52-week cadence. The multiplicative scaling of seasons over time is sampled to be either exactly 1 with a probability of 0.7 or proportional to $exp(a * n)/(1 + exp(a * n))$ with a probability of 0.3, where n indexes the number of waves and a is drawn from the normal distribution with mean 0 and standard deviation 0.5. These seasonal time series are then scaled up to the 4-week cadence (as opposed to their original weekly cadence) with probability 0.5.

S1.1.5 Adding noise to combined case wave time series

All time series outputted from the methods described above are originally between 0 and 1. In order to transform these time series to counts space and/or add observation noise, all time series go through the following additional processing:

1. draw a new time series from a Beta distribution with parameters $\alpha * ts$ and $\alpha(1 - ts)$, where ts is the original synthetic time series and $log(\alpha)$ is drawn from a Unif$(log(50), log(1000))$ distribution

2. randomly sample whether the series will correspond to counts or proportions

3. if a proportion, the time series is rescaled such that the new time series is between 0 and π, where $log(\pi)$ is drawn from a Unif$(log(0.0005), log(0.25))$ distribution

4. if a count, multiply the time series by a population size obtained by rounding the square of a Unif$(2e5, 1e8)$ draw.

5. Optionally, pad the start and the end of the time series with zeros. We implemented this for our seasonal time series generation to ensure smooth seasonal transitions.

S1.2 Further Analysis of sMOA Prediction Interval Coverage

We calculate the sMOA forecast for late November 2021 to early June 2022 for the four most populous US States in Fig C, and compare these to the ForecastHub ensemble model ('4-week ensemble'), the ForecastHub trained ensemble model ('trained ensemble'), and the persistence model ('baseline') as references for reasonable forecasts. Unlike the forecast examples in the main text, the "as-of" data are available for these dates, so the data used to build these three comparison models is the same as that used for sMOA.

As expected from the analysis of coverage, the prediction intervals for sMOA are tighter later on in the pandemic as more information is available to learn the dispersion parameter within the Negative Binomial model. We point out that the surge of cases was significantly overshot by the 4-week ensemble and the 4-week trained ensemble, but caught by sMOA and the baseline model.

S1.3 Analysis of Synthetic Data Coverage

As claimed in the main paper, the synthetic data library is developed in a way that is meant to be comprehensive and disease-general. In this section, we examine whether "comprehensive" in this context correlates with when the synthetic data "cover" real data in terms of some distance metric. For the purposes of this analysis, we use data on Chikungunya [5], Dengue Fever [6,

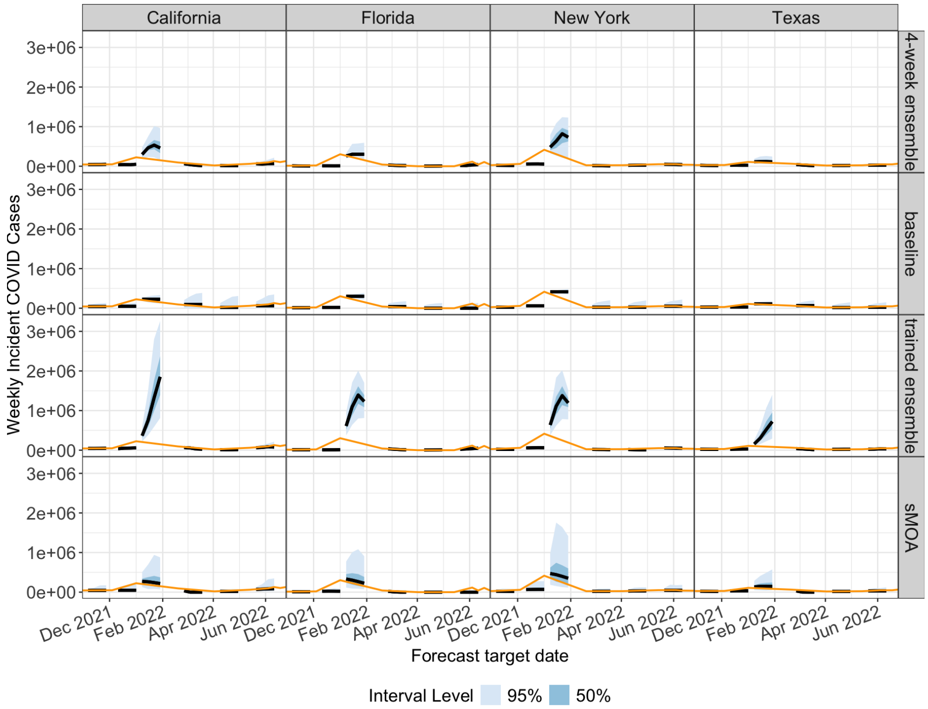

Fig C: Demonstration of sMOA forecasts from mid-November 2021 to early June 2022.

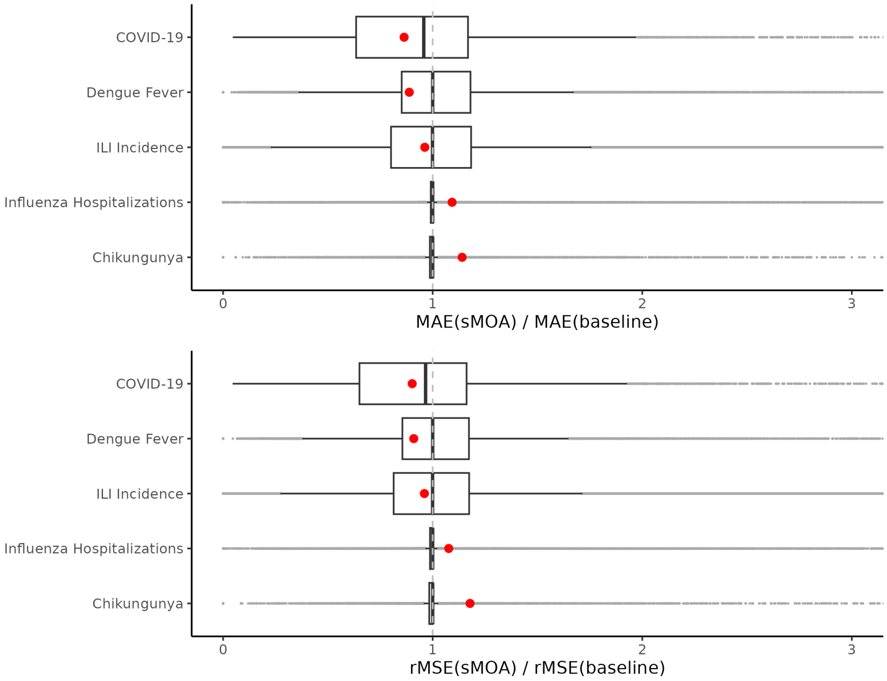

Fig D: Ratios of performance of sMOA against a baseline persistence model. Red points are calculated using average MAE or rMSE across all forecasts made for a given dataset, and boxplots are calculated using MAE or rMSE for individual forecast dates. Gray points denote outliers. Values to the left of the vertical dotted line correspond to instances where sMOA out-performed the baseline persistence model.

Disease	Time Period	Frequency of Samples	Number of Included Subregions
Chikungunya (Brazil)	2013-2022	Weekly	27
Dengue Fever	1990-2022	Weekly	24
ILI (US)	1997/2010-2023	Weekly	74
Influenza Hospitalizations (US)	2020/2021-2024	Daily/Weekly	55

Table A: Metadata for diseases used for the coverage analysis.

7, 8, 9], Influenza-like illness (ILI) incidence proportions [10], and Influenza hospitalizations [11]. For comparison, we also present results for COVID-19 using dates with available COVIDHub–Baseline model forecasts. The dates and number of regions considered for each of these diseases are outlined in Table A.

Prior to investigating the coverage of the synthetic data, we provide a brief analysis of the forecasting capabilities of sMOA on each of the five sets of additional disease data introduced in Table A. Since, unlike in our full COVID analysis in the main paper, a wealth of comparison forecasting models were not available for all of these diseases, we compare sMOA at a 4-week forecasting horizon against a basic persistence model, where the 4-week ahead forecasts are simply the last observed value.

We visualize the forecasting performance of sMOA on these five sets of disease data compared against a basic persistence model in Fig D, using both MAE and root mean squared error (rMSE). For each, we calculate the ratio between the sMOA MAE or rMSE and the corresponding values using the persistence model, where values less than 1 indicate that sMOA outperformed the persistence model. These ratios are calculated for each forecast date (summarized by boxplots), and the red points are calculated such that both the numerator and denominator of the ratio were calculated using all forecasts dates for that dataset. This figure shows that sMOA outperformed a basic persistence model on average for ILI, Dengue Fever, and COVID-19 but performed worse on Chikungunya and Influenza Hospitalizations *on average*. Notably, the distribution of ratios by forecast date is heavily right skewed for both Influenza Hospitalizations and Chikungunya. This is primarily caused by "exploding" sMOA forecasts; the average performance for Influenza Hospitalizations and Chikungunya may be improved by building in guards against exploding forecasts. The median performance for both Influenza and Chikungunya across all forecast horizons indicated a slight benefit to using sMOA over a persistence model. In the following two sections, we will investigate whether the relative performance of sMOA across datasets correlates with the coverage in the synthetic data.

This analysis was done *after* creating the synthetic data using the methods above; at no point was it used to modify the synthetic data to better cover these diseases. While "improving the coverage" of the synthetic data may improve

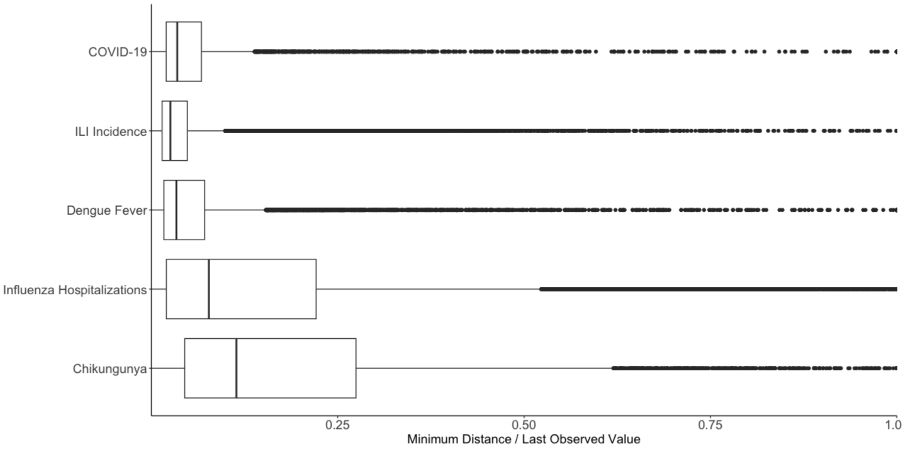

Fig E: For each forecasting instance for each disease, the distance between the k most recently observed data values and the closest synthetic data segment is divided by the last observed value. Boxplots in this figure are created from every such ratio for each disease.

the forecasting capabilities of sMOA, this would be counter to the primary aims of this paper. Updating the synthetic data to better cover these diseases would amount to training sMOA for these diseases, and we would therefore no longer be able to assess how well this method does for emerging diseases. The application in the main paper, and the analysis here, should serve to show how surprisingly well the intentionally *ad hoc* methods for created a synthetic data library in Section S1.1 perform. This being said, the authors wish to expand the synthetic data library in future papers. Ideas in this direction include using Agent Based Models and performing a leave-one-out experiment where data from other diseases are added to the synthetic data library.

S1.3.1 Distance-based Analysis of Synthetic Data Coverage

In Fig E, we visualize the normalized distances between the real data segments and the closest synthetic data segments for each disease, region, and forecast horizon; boxplots are calculated by disease. The distance metric for this visualization is minimum absolute point-wise distance. The normalization performed takes each minimum distance value and divides it by the most recently observed data value in the real data segment. This figure shows that the minimum normalized distance for each disease correlates with the persistence model comparison in Fig D; the diseases for which the minimum distance is the smallest (COVID-19, ILI, and Dengue) are also diseases where sMOA outperformed a persistence model.

While this is not decisive evidence that reducing the distance between the closest synthetic data segment and the real data for Influenza hospitalizations

and Chikungunya would improve forecasting performance, it does encourage future investigations into broadening the synthetic data library.

In the next section, we will visualize a few of these diseases with COVID using the Uniform Manifold Approximation and Project technique from [12].

S1.3.2 Uniform Manifold Approximation and Projection for Dimension Reduction

Visualizing millions of k-dimensional data objects (the segments in the synthetic data library) in a way that is informative is a difficult task that requires methodology beyond simply graphing the segments. The methodology used here is the Uniform Manifold Approximation and Projection (UMAP) method from [12]. The UMAP method creates a high-dimensional graph structure that represents the local relationships between the high-dimensional points, then projects this graph to a lower-dimensional space (here, in two dimensions) in a way that well-represents this local structure. UMAP is particularly good at visualizing which points are close to one another in a high-dimensional space; points that are drawn close to one another in the two-dimensional visualization correspond to high-dimensional points that are also close to one another. For a more in-depth, yet surprisingly readable, explanation of UMAP, see [13].

We visualize the synthetic data with the COVID-19 data from the main paper, Dengue Fever, ILI Incidence, Influenza Hospitalizations, and Chikungunya in Fig F. For the purposes of this visualization, we only use the first k values of the segments in both the synthetic data and the given real data set. These values correspond to the data that are matched in the MOA look-up procedure for the instance of sMOA used in the main paper.

Fig F suggests that the synthetic data well-cover COVID-19, Dengue Fever, and ILI Incidence, and the data potentially fail to robustly cover Influenza Hospitalizations and Chikungunya. This experiment is in line with the intuition that sMOA performs best for diseases that are well-covered by the synthetic data library, and a lack of coverage may point to poor overall performance (at least when compared to a persistence model). For example, sMOA was outperformed by a persistence model on Influenza Hospitalizations and Chikungunya. Those two outbreaks were largely composed of lower count time series. As can be seen in the right column of Fig F, low count time series are poorly represented in the synthetic library, creating a mismatch between the real and synthetic time series. Future improvements of sMOA might investigate ways for the synthetic data library to represent a broader range of real data dynamics, without being so general as to over-represent dynamics that one would not expect an emerging disease to reasonably exhibit.

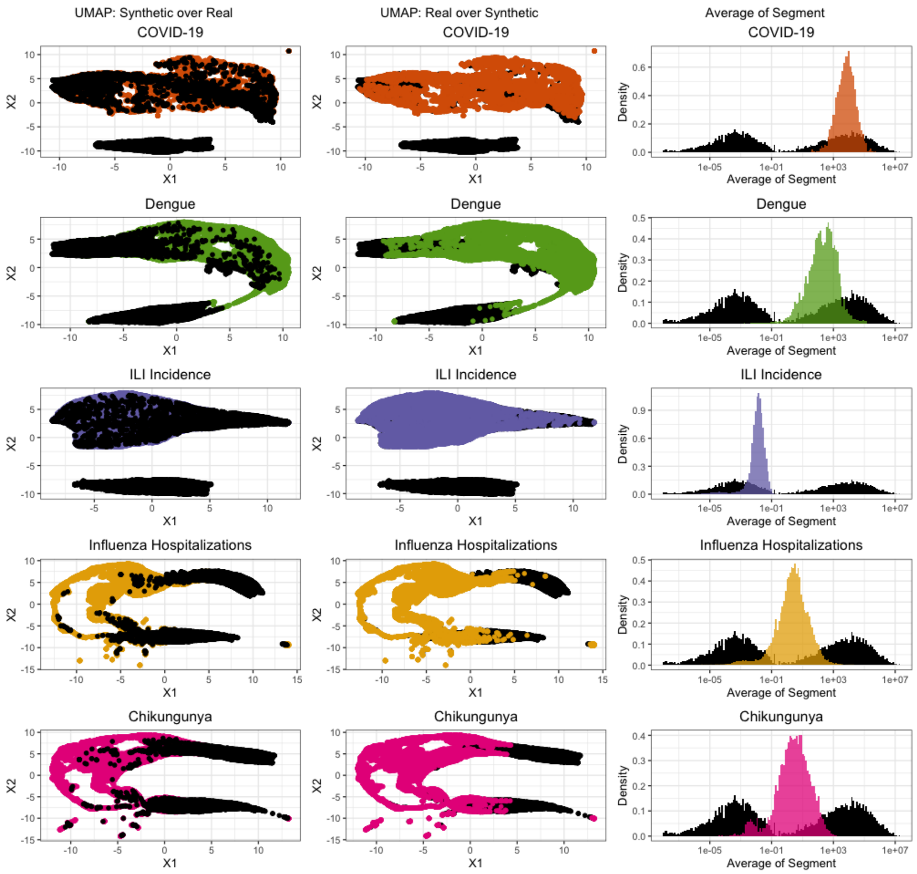

Fig F: Synthetic data library compared to real data using the UMAP method. Black points are the synthetic data, colored points are the points for each specific disease. The far left column plots the synthetic data on top of the real data; the middle column plots the real data on top of the synthetic data. The right column shows the distribution of time series segment averages; the black histograms are bimodal because half the segments are proportions and half are counts.

References

[1] Linka K, Peirlinck M, Kuhl E. The reproduction number of COVID-19 and its correlation with public health interventions. medRxiv. 2020;doi:10.1101/2020.05.01.20088047.

[2] Biggerstaff M, Cauchemez S, Reed C, Gambhir M, Finelli L. Estimates of the reproduction number for seasonal, pandemic, and zoonotic influenza: a systematic review of the literature. BMC Infectious Diseases. 2014;14(1):480. doi:10.1186/1471-2334-14-480.

[3] Díaz-Brochero C, Cucunubá ZM. Epidemiological findings, estimates of the instantaneous reproduction number, and control strategies of the first Mpox outbreak in Latin America. Travel Medicine and Infectious Disease. 2024;59:102701. doi:https://doi.org/10.1016/j.tmaid.2024.102701.

[4] Liu Y, Lillepold K, Semenza JC, Tozan Y, Quam MBM, Rocklöv J. Reviewing estimates of the basic reproduction number for dengue, Zika and chikungunya across global climate zones. Environmental Research. 2020;182:109114. doi:https://doi.org/10.1016/j.envres.2020.109114.

[5] de Souza WM, de Lima STS, Simões Mello LM, Candido DS, Buss L, Whittaker C, et al. Spatiotemporal dynamics and recurrence of chikungunya virus in Brazil: an epidemiological study. Lancet Microbe. 2023;4(5):e319–e329. doi:10.1016/S2666-5247(23)00033-2.

[6] Clarke J, Lim A, Gupte P, Pigott DM, van Panhuis WG, Brady OJ. A global dataset of publicly available dengue case count data. Scientific Data. 2024;11(296):1–14. doi:10.1038/s41597-024-03120-7.

[7] Sharp TM, Hunsperger E, Santiago GA, Muñoz-Jordan JL, Santiago LM, Rivera A, et al. Virus-Specific Differences in Rates of Disease during the 2010 Dengue Epidemic in Puerto Rico. PLOS Neglected Tropical Diseases. 2013;7(4):1–9. doi:10.1371/journal.pntd.0002159.

[8] Forshey BM, Guevara C, Laguna-Torres VA, Cespedes M, Vargas J, Gianella A, et al. Arboviral Etiologies of Acute Febrile Illnesses in Western South America, 2000–2007. PLOS Neglected Tropical Diseases. 2010;4(8):1–14. doi:10.1371/journal.pntd.0000787.

[9] Stoddard ST, Wearing HJ, Jr RCR, Morrison AC, Astete H, Vilcarromero S, et al. Long-Term and Seasonal Dynamics of Dengue in Iquitos, Peru. PLoS Negl Trop Dis. 2014;8(7):e3003.

[10] CDC U. FluView Dashboard; 2025. Available from: https://gis.cdc.gov/grasp/fluview/fluportaldashboard.html.

[11] Ray EL, Wang Y, Wolfinger RD, Reich NG. Flusion: Integrating multiple data sources for accurate influenza predictions; 2024. Available from: https://arxiv.org/abs/2407.19054.

[12] Healy J, McInnes L. Uniform manifold approximation and projection. Nature Reviews Methods Primers. 2024;4(1):82. doi:10.1038/s43586-024-00363-x.

[13] McInnes L. How UMAP Works; 2018. Available from: https://umap-learn.readthedocs.io/en/latest/how_umap_works.html.

Contents

S1 Text **1**
- S1.1 Synthetic Data Generation 1
  - S1.1.1 Combining Multiple Case Waves 3
  - S1.1.2 Susceptible-Infected-Recovered “Rollercoaster” Model . . 4
  - S1.1.3 Susceptible-Infected-Recovered “Wiggle” Model 4
  - S1.1.4 Seasonal Model 4
  - S1.1.5 Adding noise to combined case wave time series 5
- S1.2 Further Analysis of sMOA Prediction Interval Coverage 5
- S1.3 Analysis of Synthetic Data Coverage 5
  - S1.3.1 Distance-based Analysis of Synthetic Data Coverage . . . 9
  - S1.3.2 Uniform Manifold Approximation and Projection for Dimension Reduction 10
